# Sequencing and de Novo Assembly of Abaca (*Musa textilis* Née) var. Abuab Genome

**DOI:** 10.3390/genes12081202

**Published:** 2021-08-02

**Authors:** Leny Calano Galvez, Rhosener Bhea Lu Koh, Cris Francis Cortez Barbosa, Jayson Calundre Asunto, Jose Leonido Catalla, Robert Gomez Atienza, Kennedy Trinidad Costales, Vermando Masinsin Aquino, Dapeng Zhang

**Affiliations:** 1 Philippine Fiber Industry Development Authority (PhilFIDA), PCAF Bldg, DA Compound, Diliman, Quezon City 1101, Philippines; research.cfb@gmail.com (C.F.C.B.); jcasunto.philfida@gmail.com (J.C.A.); jlcatalla@yahoo.com (J.L.C.); rgatienza@philfida.da.gov.ph (R.G.A.); ktcostales@yahoo.com (K.T.C.); 2 National Institute of Molecular Biology and Biotechnology, University of the Philippines Diliman, Quezon City 1101, Philippines; rlkoh@up.edu.ph (R.B.L.K.); vmaquino@mbb.upd.edu.ph (V.M.A.); 3 Sustainable Perennial Crops Laboratory, USDA-ARS, Beltsville, MD 20705, USA; dapeng.zhang@usda.gov

**Keywords:** *Musa textilis* Née, fiber crop, Manila hemp, Abuab, NGS, whole genome sequencing, de novo assembly, Musa spp.

## Abstract

Abaca (*Musa textilis* Née), an indigenous crop to the Philippines, is known to be the source of the strongest natural fiber. Despite its huge economic contributions, research on crop improvement is limited due to the lack of genomic data. In this study, the whole genome of the abaca var. Abuab was sequenced using Illumina Novaseq 6000 and Pacific Biosciences Single-Molecule Real-Time Sequel. The genome size of Abuab was estimated to be 616 Mbp based on total k-mer number and volume peak. Its genome was assembled at 65× depth, mapping 95.28% of the estimated genome size. BUSCO analysis recovered 78.2% complete BUSCO genes. A total of 33,277 gene structures were predicted which is comparable to the number of predicted genes from recently assembled Musa spp. genomes. A total of 330 Mbp repetitive elements were also mined, accounting to 53.6% of the genome length. Here we report the sequencing and genome assembly of the abaca var. Abuab that will facilitate gene discovery for crop improvement and an indispensable source for genetic diversity studies in Musa.

## 1. Introduction

One of the major agricultural export commodities of the Philippines is the abaca (*Musa textilis* Née), a fiber crop widely distributed in the humid tropics, and is known to be indigenous to the Philippines [1,2]. About 85% of the world market consumption for abaca fiber is supplied by the Philippines which generates a total of US$111.5 M earnings in 2018 [3]. The huge economic value of abaca is a driving force in the Philippine agriculture with an industry that supports the livelihood of nearly 1.5 M Filipinos including 122,758 farmers who cultivate a total of 180,302 hectares of abaca [3]. The abaca plant is a close relative of the banana, belonging to the family Musaceae of the order Zingibareles [2]. The Musaceae family is further divided into two genera, the Musa and Ensete which are known for their edible fruits. Unlike most of the Musa spp., the fruits of *Musa textilis* are inedible as they contain numerous large black viable seeds (20–200 seeds per fruit) [4]. The abaca plant can reach up to 10 feet with a base root stock of up to 20 inches in diameter and is slightly narrower than that of a banana. Most notable are the leaf sheaths arising from the base root stock which form the pseudostem from which the useful fibers are extracted [4].

Demand for abaca fiber is generally attributed to its fiber’s mechanical properties and high-resistance to salt-water damage [1]. Abaca is considered to be the strongest of all the natural fibers and has been largely used for the production of cordage, textile and paper products [1,2]. As abaca fiber is considered a renewable bioresource, it has recently been utilized as an alternative raw material for fiber composites used for infrastructure, automobile body parts and aerospace materials [5,6,7]. These characteristics make abaca the fiber of choice for various industrial applications; thus, increased fiber production is needed to meet the world market demand.

However, abaca fiber production is hindered by low fiber yield due to biotic and abiotic factors [8]. The prevalence of viral diseases such as abaca bunchy top virus (ABTV), banana bunchy top virus (BBTV), banana bract mosaic virus (BBrMV), cucumber mosaic virus (CMV) and sugarcane mosaic virus (SCMV) [9,10,11,12,13] causes significant crop losses as the viruses negatively impact abaca fiber yield and quality [8,14]. Furthermore, natural calamities in the Philippines cause huge economic losses to abaca production that for the period 2000 to 2010 losses due to typhoons, floods and drought amounted to a total of US$9.55 million [15].

Efforts have been done to mitigate the effects of these debilitating factors while increasing abaca fiber production. Continuous characterization of germplasm collections are being done in search for high yielding and disease tolerant varieties. Currently, there are three National Seed Industry Council (NSIC)-registered abaca varieties cultivated for abaca fiber production, namely Abuab, Inosa and Tangongon [16]. These varieties are being recommended to specific regions in the country for yield and adaptability considerations. The variety Abuab is recommended to the Bicol Region, Inosa to the Visayas Region and Tangongon for Mindanao Region. Due to the location specificity nature of abaca, these varieties would perform differently outside their recommended regions depending on the agro-climatic conditions. Additionally, some varieties have been observed to have selective resistance to pathogens such as Inosa and Tangongon against *Fusarium oxysporum* var cubense [17,18].

To mitigate problems in abaca virus diseases, PhilFIDA developed abaca virus detection technologies applicable in both laboratory research settings [19,20,21,22] and point-of-care diagnostics [20,23,24] for effective disease management. Moreover, one of the major undertakings of PhilFIDA is the implementation of the Abaca Disease Management Program (ADMP) that aims to eradicate and rehabilitate disease-infected abaca areas. Insufficient as a solution; this must be coupled with a more sustainable strategy like the introduction of a disease resistant and high yielding variety. Relative to the genetic improvement of commercially cultivated abaca, the need for characterizing different abaca varieties especially their molecular characteristics becomes imperative.

The genetic pool of abaca is highly diverse as indicated by a high Shannon diversity index in several Philippine genebanks that could be a vast resource for breeding of elite varieties [8]. Currently, there are no publicly available assembled draft abaca genomes for genome comparison studies of these varieties. On the other hand, there are draft genomes available for other Musa spp. such as *Musa acuminata* [25,26,27], *Musa balbisiana* [28,29], *Musa itinerans* [30] and *Musa schizocarpa* [31]. Although these Musa spp. are under the same genera as with abaca, the genus Musa is so diverse that it is further divided into two sections: Musa (where edible bananas belong) and Callimusa (where abaca belongs) [32]. Availability of the abaca draft genome will facilitate the identification of specific genes responsible for agronomically and economically important traits of abaca such as disease resistance, fiber quality, fiber yield, stooling capacity and environmental stress resistance/tolerance.

In this paper, we present an assembled whole genome sequence of abaca variety Abuab. This draft genome is an important tool for maximizing gene discovery for agronomic performance, fiber quality and disease-resistance, as well as for molecular marker development, and routine marker-assisted breeding applications. Moreover, it is an indispensable resource for Musa spp. genetic diversity studies.

## 2. Materials and Methods

### 2.1. Abaca Sample Collection, DNA Extraction and Library Preparation

Abaca plantlet var. Abuab (NSIC 2017 Mt 001) was obtained from the Albay Tissue Culture Laboratory. Total DNA was extracted using the cetyl trimethylammonium bromide with 0.3% β-mercaptoethanol protocol [33].

Pacific Biosciences Single-Molecule Real-Time Sequel (PacBio SMRT) and Illumina Novaseq whole genome libraries were prepared using 20 kb SMRTbell Express Kit (Pacific Biosciences, Menlo Park, CA, USA) and TrueSeq Nano DNA Kit (Illumina, San Diego, CA, USA), respectively. Library construction was performed by random fragmentation of DNA, followed by ligation of 5’ and 3’ adapters. The adapter-ligated fragments were then amplified through PCR, and were purified through gel purification.

### 2.2. Sequencing and Assembly

Paired-end sequencing was performed in the NovaSeq 6000, respectively. The sequencing data was converted into raw data, which subsequently underwent pre-processing measures. The raw reads went through quality control, using FastQC (v.0.11.5), through calculation of total bases, total reads, GC content, overall quality of the reads generated and basic statistics. To reduce bias in the analysis, the raw data underwent adapter trimming and quality filtering using Trimmomatic (v.0.38). Pre-processing also included search and removal of integrated viral sequences and organelle (mitochondria and chloroplast) DNA.

After conducting pre-processing, k-mer analysis was performed using Jellyfish (v.2.2.10) [34] and GenomeScope [35]. Data on k-mer coverage, heterozygosity and estimated genome size were obtained.

De novo assembly was done using Platanus-allee 2.2.2 [36] utilizing the filtered sequences generated through Illumina. To improve the quality of the assembly, scaffolding and gap-filling using the PacBio Sequel data were performed using PBJelly2 v.15.8.24 [37]. A best-kmer for the sequenced genome was selected using statistics from the assembly results. RepeatMasker 4.1.1 (http://repeatmasker.org) (Accessed on 7 January 2021) was used to mask repetitive elements among the scaffolds. Moreover, *Musa acuminata* repeats were also masked using—species option of the RepeatMasker.

The assembled abaca genome was validated through BUSCO (v.3.0) analysis [38] and self-mapping analysis. BUSCO analysis involved evaluation of the assembled genomes on the basis of evolutionary-informed expectations of the gene contents using the eukaryota_odb9 lineage dataset (number of species: 100, number of BUSCOs: 303). Self-mapping analysis was performed by mapping the Illumina reads against the scaffolds to provide information on assembly quality.

### 2.3. Gene Prediction and Annotation

The genome was annotated using the MAKER pipeline incorporating ab initio predictions, CDS and protein sequences of single copy BUSCOs and transcripts and protein sequences of *Musa acuminata*. This process involved construction of a HMM model using SNAP (version 2006-07-28), which was used to predict genes via MAKER 2.31.10. To identify the putative functions of the predicted genes, all gene models were aligned using blastp (with a threshold of E-value < 1 × 10^−5^) against GenBank non-redundant, UniProt (v.201806), Gene Ontology (GO) database, InterPro (v.69.0), Protein family (Pfam) (v.31.0), CDD (v.3.16), TIGRFAMs (v.15.0), evolutionary genealogy of genes: Non-supervised Orthologous Groups (eggNOG v.4.5), Kyoto Encyclopedia of Genes and Genomes Ortholog (KEGG) databases.

### 2.4. Orthogroup and Phylogenetic Analyses of Musa textilis var. Abuab

Peptide sequences from eight plant species: *Musa acuminata*, *Musa balbisiana*, *Ensete glaucum* (*Musa nepalensis*), *Musa schizocarpa*, *Oryza sativa*, *Arabidopsis thaliana*, and *Gossypium raimondii* were retrieved from Gramene-Ensembl Biomart database [39]. Together with the *Musa textilis* var. Abuab peptide sequence, the downloaded protein sequences were filtered for their respective longest isoform for each gene. This was done using the python script provided in the CAFÉ (Computational Analysis of Gene Family Evolution) tutorial [40].

The filtered sequences were used as an input for OrthoFinder 2.5.2 [41]. Common orthologous groups among the eight species were analyzed through the generation of venn diagrams [42]. The gene tree inference of the program was set to use MAFFT 7.475 [43] and RAxML 8 [44]. The sequence search algorithm was set to use DIAMOND 2.0.9 [45]. The species tree algorithm was set to use Fast Tree 2 [46]. Remaining settings were set to the program’s default parameters. The phylogenetic tree was viewed with iTOL (Interactive Tree of Life) [47].

### 2.5. TE Repeat Analysis and Annotation

Transposable element annotation was conducted using Extensive de novo TE Annotator (EDTA) [48]. Using Perl command, EDTA software was set to run using the abuab fasta as input file. In addition, the --step option was set to ‘all’ to run the entire annotation pipeline of the software. The --sensitive option was set to ‘1’ in order for the software to detect additional TEs using EDTAs embedded RepeatModeler tool. The --anno option was set to ‘1’ to conduct whole-genome annotation of the TEs. Results were extracted from the *. fasta.mod.EDTA.TEanno.sum files and annotation statistics were summarized and visualized using Microsoft Excel.

### 2.6. Data Availability

The assembled Abaca var. Abuab sequence, transcript and protein sequences and genome annotation GFF file has been stored in the Data Dryad Digital Repository (https://datadryad.org/stash/) (uploaded on 1 June 2021) under the DOI 10.5061/dryad.95x69p8kt. which is publicly accessible at https://datadryad.org/stash/share/Yk6Ls1qw7WQts4zl03iPEchuiw6kMKBJBy6Oa1-JN00.

## 3. Results and Discussion

### 3.1. Assembled Sequences of Abuab

A recently sequenced *Musa textilis* genome was reported by Sambles et al. [49]; however, the study only employed Illumina HiSeq sequencing reads which generated 23× coverage depth. In this study, the whole genome sequence of abaca var. Abuab was generated using Illumina NovaSeq and PacBio SMRT technologies. Illumina is a second-generation sequencing platform which involves clonal amplification of adaptor-ligated DNA [50]. This technology has the lowest error rate, highest throughput, and is the cheapest NGS platform. NovaSeq 6000, the most recently released Illumina system, can generate 20 billion reads per run, and has a maximum paired read length of 150 bp [51]. PacBio SMRT is a third generation platform that has the ability to sequence single DNA molecules, and uses hairpin adaptors to generate a closed ssDNA template, i.e., SMRTbell (SMRT is an abbreviation of single molecular real-time sequencing) [52] Sequel, which is its latest instrument, can deliver up to 370,000 reads. Among other platforms, PacBio sequencing technology generally produces longer sequencing reads than Illumina paired-end reads but also contains high error rates [51]. These technologies were used to generate a draft genome of abaca var. Abuab. The Abuab variety was selected for sequencing as it is commercially grown by abaca farmers due to its high fiber recovery and uniform morphology across regional locations. Moreover, it is one of the first registered abaca varieties. Elucidation of the draft genome shall serve as important tools for improvement of agronomic performance and fiber quality in abaca.

**Figure 1:**
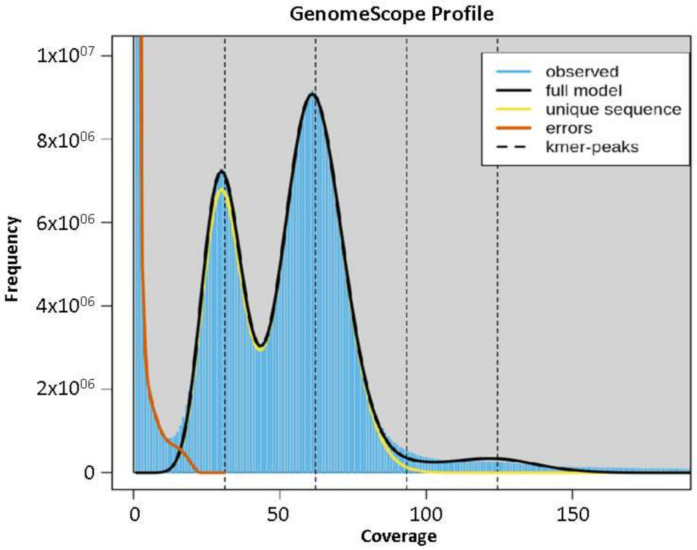
K-mer graph of abaca var. Abuab genome assembly.

The Abuab genome was estimated to have a genome size of 616 Mbp based on total k-mer number and volume peak (Figure 1), and a heterozygosity rate of 1.111. The estimated genome size of abaca is higher than reported genome sizes of other Musa species such as *Musa acuminata* (600 Mbp) [26], *Musa balbisiana* (554 Mbp) [29], *Musa itenerans* (462 Mbp) [30] and *Musa schizocarpa* (587 Mbp) [31]. Utilizing Illumina reads with gap-filling steps using the PacBio Sequel data as the assembly strategy and using 99.71% total reads, in mapping to scaffolds were performed to construct the draft genomes.

### 3.2. Characteristics of Final Genome Assembly

The final assembly (after scaffolding, gap-filling and repeat masking) returned an abaca genome with an assembled length of 587 Mbp covering 95.28% of the estimated genome size, and having a scaffold N50 value of 47,291 (Table 1). Benchmarking Universal Single Copy Ortholog (BUSCO) analysis using 100 species in the eukaryota_odb9 database as reference revealed the final assembled abaca genome to contain 237 (78.2%) complete BUSCOs (consisting of 173 single copy and 64 duplicated complete BUSCOs), 20 fragmented BUSCOs (6.6%) and 46 missing BUSCOs (15.2%). The high percentage of complete BUSCOs indicates high level completeness of the assemblies (Table 1). The assembled abaca genome is composed of 40.23% GC content and also possessed 330 Mbp repeat length, accounting to 53.6% of the genome length (Table S1).

The quality of the Abuab genome assembly was compared with the genome assembly of related Musa species. In terms of assembly quality statistics, the Abuab genome has lower N50 values but comparable percent complete BUSCOs (Table 1). The relatively low N50 values of the assembled abaca genomes were compensated by a high level of scaffolding, therefore resulting in comparable assembly completeness and percent coverage. Among the compared genome assemblies, the *M. balbisiana* genome assembled [29] possesses the highest quality in terms of contiguity, completeness and coverage of the expected genome size. Like this study, Wang et al. [29] utilized both Illumina and Pacbio platforms for sequencing of the *M. balbisiana* genome. However, their study utilized 113× Pacbio reads and 166× Illumina reads to generate the *M. balbisiana* genome, which are higher than the 65× depth for abaca Illumina assembly. Nevertheless, the abaca genome covered the highest portion of the Musa genome, and is of satisfactory quality. This assembled abaca genome can therefore provide a good reference for downstream genomic studies, marker development and re-sequencing projects.

### 3.3. Gene Prediction, Functional Annotation and Classification of the Assembled Abaca Unigenes

MAKER is a user-friendly genome annotation pipeline tool heavily used in identifying ESTs, repeats, and proteins, and is also used in conducting gene predictions in a genome. MAKER can also be pre-trained to use specific gene prediction algorithms in order to output finer models and accurate statistics of the analysis [53]. SNAP (Semi-HMM-based Nucleic Acid Parser) is a flexible gene finder tool capable of processing accurate ab initio gene prediction of various organisms. SNAP can also be trained to conduct de novo gene predictions of newly sequenced genomes by generating HMM (hidden Markov models).

**Figure 2:**
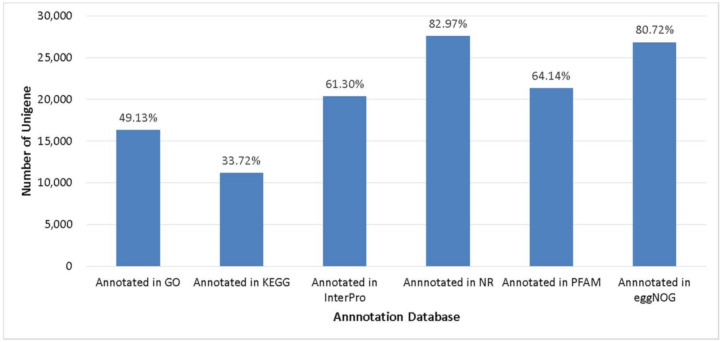
Unigenes annotated in several databases.

Gene structures were predicted using MAKER 2.31.10 and were narrowed using transcripts and protein sequences of *Musa acuminata*. A total of 33,277 genes in abaca var. Abuab genome were detected and retained (Table 2). Analysis of the 50 first plant genomes sequenced showed the majority of plant genomes have between 20,000 to 94,000 genes with a median predicted gene count of 32,605 [54]. Comparison of the number of predicted genes in abaca compared to other Musa spp. showed that *M. balbisiana*, *M. itinerans* and *M. schizocarpa* were the closest to *M. textilis* (Table 2). Predicted gene models were aligned against six databases with BLASX against NR database (27,609 unigenes) and eggNOG databases (26,861 unigenes) showing the highest gene annotation rate (Figure 2).

**Figure 3:**
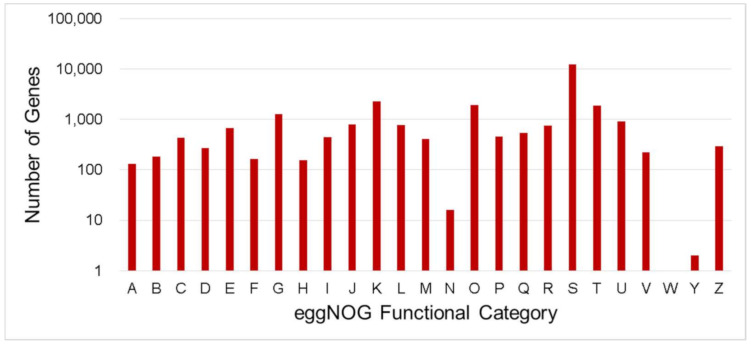
Number distribution of eggNOG annotation of Unigenes related to A–Z. A—RNA processing and modification; B—Chromatin structure and dynamics; C—Energy production and conversion; D—Cell cycle control, cell division, chromosome partitioning; E—Amino acid transport and metabolism; F—Nucleotide transport and metabolism; G—Carbohydrate transport and metabolism; H—Coenzyme transport and metabolism; I—Lipid transport and metabolism; J—Translation, ribosomal structure and biogenesis; K—Transcription, L—Replication, recombination and repair; M—Cell wall/membrane/envelope biogenesis; N—Cell motility; O—Posttranslational modification, protein turnover, chaperones; P—Inorganic ion transport and metabolism; Q—Secondary metabolites biosynthesis, transport and catabolism; R—General function prediction only; S—Function unknown; T—Signal transduction mechanisms; U—Intracellular trafficking, secretion, and vesicular transport; V—Defense mechanisms; W—Extracellular structures; Y—Nuclear structure; Z—Cytoskeleton.

GO annotation is a unified classification or representation system that provides a standardized term or vocabulary for assigning functions of genes and gene products of uncharacterized sequences [55]. Out of the 33,277 gene products predicted from the assembled abaca genome, a total of 16,350 protein sequences were successfully annotated to the GO database with three main ontologies: cellular component, molecular function and biological process (Figure 3). “Cell” and “cell part” were the most abundantly represented subcategories under cellular component. “Catalytic activity” and “binding” were the top two represented category in the molecular function subcategory. Within the biological process subcategory, “metabolic process” and “cellular process” had the largest number of unigenes.

EggNOG-mapper is an annotation tool for functional annotation of uncharacterized sequences which uses orthology relationships, gene evolutionary histories and functional annotations from the eggNOG database [56]. In total, 26,861 unigenes annotated by eggNOG were classified into 25 categories (Figure 3). The top five (5) number of unigenes were classified as “Function unknown” (12,389 and 45.35%), followed by “Transcription” (2268 and 8.30%), “Posttranslational modification, protein turnover, chaperones” (1917 and 7.02%), “Signal transduction mechanisms (1859 and 6.80%) and “Carbohydrate transport and metabolism” (1269 and 4.65%).

### 3.4. Comparative and Evolutionary Genomics

**Figure 4:**
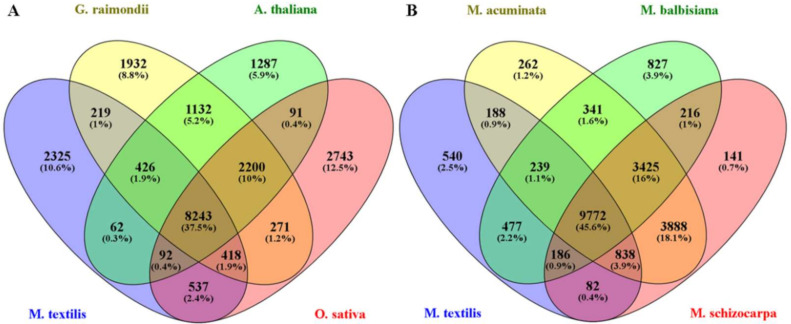
Venn diagram showing the number of orthologous groups. (**A**) The common and unique orthologous groups among *M. textilis*, *G. raimondii*, *A. thaliana* and *O. sativa*. (**B**) The common and unique orthologous groups among *M. textilis*, *M. acuminata*, *M. balbisiana* and *M. schizocarpa*.

OrthoFinder was used as another approach to evaluate the assembly completeness of M. textilis var. Abuab based on sequence similarity. Using protein sequences from eight plant species, 286,438 genes were analyzed with 254,230 (88.8%) genes assigned to orthologous protein groups (orthogroups) (Table S2). These genes were assigned to a total of 28,109 orthogroups. OrthoFinder analysis showed that *M. textilis* had 24,191 genes assigned to orthogroups which represents 87.6% of the total protein sequences used for the analysis (Table S3). *M. textilis* also had 3418 unclustered genes which showed comparable and even lower values from the remaining seven plant species (Table S3). The 24,191 genes families identified in *M. textilis* were assigned to 12,322 orthogroups (Table S3). A 4-way comparison of the *M. textilis* species with *A. thaliana*, *G. raimondii* and *O. sativa* showed 8243 gene families were shared among them and 2325 gene families were unique to *M. textilis* (Figure 4A). More in depth analysis of common orthogroups showed that *M. textilis* had 9306 and 9290 overlapping orthologous protein groups with *G. raimondii* and *O. sativa*, respectively (Table S4). This indicates that more gene families are shared between abaca and *G. raimondii* and between abaca and *O. sativa* as compared to *A. thaliana*. A 4-way comparison of the four Musa species showed 9772 gene families were shared among them and 540 were found to be unique to *M. textilis* (Figure 4B). Out of the three Musa species sequences compared, M. acuminata had the greatest number of shared orthogroups with abaca wherein they had 11,037 overlapping orthologous protein groups (Table S4) which suggests higher similarity for these two Musa species.

Orthofinder’s multiple sequence alignment, using MAFFT and RAxML, generated a species tree comprising the *Musa textilis* var. Abuab peptide sequence, and the seven downloaded peptide sequences from Gramene-Ensembl Biomart. A total of 2507 orthogroups were used for the multiple sequence alignment.

**Figure 5:**
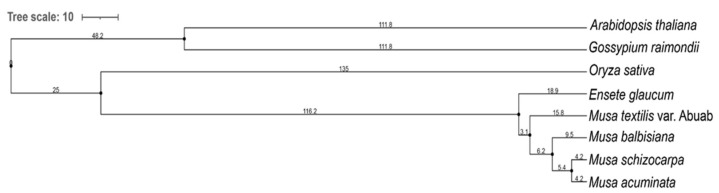
Phylogenetic tree of *Musa textilis* var. Abuab, four other Musa spp., *Oryza sativa*, and two outgroup species, *Arabidopsis thaliana* and *Gossypium raimondii* using OrthoFinder 2.5.2 Multiple Sequence Alignment, set to use MAFFT 7.475 and RAxML.

The phylogenetic tree (Figure 5) showed distinct clade differentiation between the Musa sp., *O. sativa* and the two outgroup species, *A. thaliana* and *G. raimondii*. The outgroup species have more ancestral orthologous genes compared to the Musa sp., and that the latter diverged more recently at a closer ontology period.

According to the tree scale values (branch length, BL) of Musa sp., *E.glaucum*, also known as *Musa nepalensis*, has the highest ancient ontology among the five Musa sp. analyzed (BL = 18.9). On the other hand, both *M. schizocarpa* and *M.balbisiana* may have diverged recently (BL = 4.2). The five Musa sp. included in the phylogenetic tree have been historically subjected to selection forces, geographical isolation/boundaries, and mode of propagation [57,58,59]. These factors affect the clade differentiation reflected in the generated phylogenetic tree.

### 3.5. TE Repeat Analysis and Annotation

Extensive de novo TE Annotator (EDTA) classified the transposable elements into five (5) TE classifications (LTR, TIR, non-LTR, non-TIR and repeat regions) and unknown TEs. EDTA mapped a total of 497,082 transposable elements, including the unknown TEs, totalling to 69,168 (13.98%). TEs are further classified into their respective superfamilies (Table 3). LTR/Copia was the most abundant TE among the superfamilies, having 171,310 counts (34.46%).

The results of TE analysis using EDTA was proportional to similar studies related to the repeat diversity of Musa species. LTR/Copia was also found to be dominant in both *M. balbisiana* var. Pahang (19.42% basepair masked) and *M. balbisiana* var. Pisang Klutuk Wulung (18.83% BP masked) [28]. In addition, a similar study on the diversity analysis of Musaceae Family using genome proportions also reported abundance of the LTR/Copia in *M. acuminata*, *M. ornata* (28.57% BP masked), *M. balbisiana* (23.02% BP masked), *M. beccanii* (25.81% BP masked), *M. textilis* (29.02% BP masked) and *Ensete gilletti* (12.75% BP masked). LTR-retrotransposons are largely found in higher plant genomes, and mainly contribute to genome size evolution [60].

The differences in the number of identified transposable elements among the Musa spp. can be due to the degree of domestication throughout time.

## 4. Conclusions

The lack of genomic information on *M. textilis* has hindered the progression of molecular studies on the key components of fiber development, disease and stress resistance as well as the development of molecular markers to characterize different varieties of M. textilis. In this study, we have assembled the first draft genome of *M. textilis* variety Abuab based on Illumina short-read sequencing and PacBio long-read sequencing. Comparison of the de novo assembled genome of abaca to other whole genome sequences of Musa species showed comparable qualities in terms of genome length, coverage and number of coding genes identified. Annotation of the abaca var. Abuab genome showed a comparable number of gene predictions compared to closely related Musa spp. Transposable element annotation determined a large percentage of LTR/Copia among the TE superfamiles found in the abaca var. Abuab genome. The genome sequence obtained here should accelerate genomic and molecular studies of *M. textilis* and should provide a good reference draft sequence for further sequencing and genome analysis of other abaca varieties. Moreover, these results formed part of the molecular toolbox for abaca crop improvement programs and addressed the dearth of information on abaca genomics.

## Figures and Tables

**Table 1:** Comparison of statistics generated in literature for the genome assembly of different *Musa* species.

	Number of Scaffolds	N50	% Complete BUSCOS	Assembly % Coverage
*M. textilis* (This study)	48,495	47,291	78.2 ^a^	95.28
*M. balbisiana* [29]	378	5,050,000	91.3 ^b^	95.0
*M. schizocarpa* [31]	194	44,786,816	92.3 ^b^	89.4
*M. acuminata ssp. banksii* [27]	9467	435,833	95.3 ^b^	78.2
*M. acuminata ssp. burmannica* [27]	47,753	22,183	80.2 ^b^	84.4
*M. itinerans* [30]	7194	192,092	N/A	75.2
*M. acuminata* [26]	1532	3,014,384	N/A	N/A

^a^ Total BUSCO groups searched: 303. ^b^ Total BUSCO groups searched: 1440.

**Table 2:** Gene prediction results for sequenced *M. textilis* var. Abuab genome.

	*M. textilis* ^a^	*M. acuminata* ^b^	*M. balbisiana* ^b^	*M. itinerans* ^c^	*M. schizocarpa* ^d^
Number of genes	33,277	48,628	35,148	32,456	32,809
Average CDS length	1115	879	1144	1065	1127
Average exon number	5	4	5	5	5
Average exon length	228	232	284	207	N/A
Average intron length	843	927	809	613	715

^a^ Data from this study. ^b^ Data from [29]. ^c^ Data from [30]. ^d^ Data from [31].

**Table 3:** Consolidated TE annotation and distribution of abaca var. Abuab.

TE Classification	Count	TE Ratio
Long Terminal Repeats (LTR)		
Copia	171,310	34.46%
Gypsy	49,839	10.03%
Terminal Inverted Repeats (TIR)		
CACTA	21,359	4.30%
Mutator	41,992	8.45%
PIF-Harbinger	4880	0.98%
Tc1-Mariner	13,409	2.70%
hAT	17,768	3.57%
Non-LTR		
LINE-Element	4556	0.92%
Non-TIR		
Helitron	10,563	2.13%
Repeat Region	92,238	18.56%
Unknown	69,168	13.91%
TOTAL	497,082	100%

## Data Availability

The data presented in this study are openly available in the Data Dryad Digital Repository (https://datadryad.org/stash/) under the DOI 10.5061/dryad.95x69p8kt, publicly accessible at https://datadryad.org/stash/share/Yk6Ls1qw7WQts4zl03iPEchuiw6kMKBJBy6Oa1-JN00.

## Supplementary Materials

The following are available online at https://www.mdpi.com/article/10.3390/genes12081202/s1, Table S1. Statistical summary of abaca var. Abuab genome assembly. Table S2. The total Orthogroups from eight plant species. Table S3. Summary of gene family clustering. Table S4. Overlapping orthologous groups among the eight species.
