# Supplementary material for: Sequencing and de Novo Assembly of Abaca (Musa textilis Née) var. Abuab Genome
Source: Genes (Basel). 2021 Aug 2;12(8):1202. doi: 10.3390/genes12081202 (PMC8392402; doi:10.3390/genes12081202)
Supplement: Supplementary file 1 [file genes-12-01202-s001.zip › genes-1202454-supplementary.pdf]

Supplementary Tables

Table S1. Statistical summary of abaca var. Abuab genome assembly.

	**Criteria**	**Sequencing Platform**		
		**Illumina**	**PacBio**	**Illumina + PacBio**
Raw Data Statistics	Total Read Bases	49,484,985,536	8,489,255,666	
	Total Reads	327,715,136	554,475	
	Q20 (%)	97.24		
	Q30 (%)	93.19		
Filtered data statistics	Total Read Bases	44,700,877,179		
	Total Reads	296,459,834		
	GC (%)	40.23		
	Q20 (%)	98.98		
	Q30 (%)	95.65		
K-mer analysis	k-mer coverage		62.16	
	Heterozygosity		1.111	
	Genome length		616,569,438	
	Genome Repeat Length		330,319,007	
Assembly summary of contig	Number of contigs			58,121
	Contigs Sum (total number of bases in the contigs)			613,825,282
	N50		20,999	26,944
	Longest contig		116,432	275,661
	Shortest contig		50	1,000
	Average length		15,310	10,561
Assembly summary of scaffold	Number of scaffold			48,495
	Scaffolds sum			650,778,343
	N50			47,291
	Longest			389,714
	Shortest			981
	Average length			13,419
Overall mapping statistics	Total reads		296,459,834	
	Mapped reads		295,609,701 (99.71%)	
	Coverage (%)		95.28	
	Depth		65.01	
	Ins.size (Std.)		419.5 (99.30)	
	Complete<br>• Single-copy<br>• Duplicated			 173 (57.1%)<br>64 (21.1%)

BUSCO Analysis Result	Fragmented	20 (6.6%)
	Missing	46 (15.2%)
	Total BUSCO groups searched	303 (100%)

Table S2. The total Orthogroups from eight plant species.

Number of genes	Genes in orthogroups	Number of unassigned genes#[1]	Number of orthogroups	Number of species-specific orthogroups#[2]	G50#[3]	O50#[4]
286,438	254,230	32,208	28,109	6,029	13	5,967

[1]**Unassigned gene:** A gene that has not been put into an orthogroup with any other genes.
[2]**Species-specific orthogroup:** An orthogroups that consist entirely of genes from one species.
[3]**G50:** The number of genes in the orthogroup such that 50% of genes are in orthogroups of that size or larger.
[4]**O50:** The smallest number of orthogroups such that 50% of genes are in orthogroups of that size or larger.

Table S3. Summary of gene family clustering.

Species	Total genes	Genes in families	Unclustered genes#[1]	Number of orthogroups	Species-specific orthogroups#	Genes in species-specific orthogroups
M. textilis	27,609	24,191	3,418	12,322	228	648
M. acuminata	36,519	33,472	3,047	18,953	103	247
M. balbisiana	33,021	29,027	3,994	15,483	163	492
E. glaucum *(M. nepalensis)*	36,836	30,458	6,378	15,687	186	501
M. schizocarpa	32,809	31,886	923	18,548	35	86
A. thaliana	32,799	29,674	3,125	13,533	1,231	5,527
G. raimondii	46,100	41,936	4,164	14,841	1,725	7,080
O. sativa	40,745	33,586	7,159	14,595	2,358	10,524

[1] **Unclustered genes:** A gene that has not been put into an orthogroup with any other genes.

Table S4. Overlapping orthologous groups among the eight species.

	A. thaliana	***E. glaucum (M. nepalensis)***	***G. raimondii***	***M. acuminata***	***M. balbisiana***	***M. schizocarpa***	***M. textilis***	***O. sativa***
A. thaliana	13,533	10,975	12,001	10,844	10,470	10,903	8,823	10,626
E. glaucum (M. nepalensis)	10,975	15,687	11,531	14,078	13,747	14,041	10,994	11,477
G. raimondii	12,001	11,531	14,841	11,443	11,012	11,457	9,306	11,132
M. acuminata	10,844	14,078	11,443	18,953	13,777	17,923	11,037	11,348
M. balbisiana	10,470	13,747	11,012	13,777	15,483	13,599	10,674	10,929
M. schizocarpa	10,903	14,041	11,457	17,923	13,599	18,548	10,878	11,335
M. textilis	8,823	10,994	9,306	11,037	10,674	10,878	12,322	9,290
O. sativa	10,626	11,477	11,132	11,348	10,929	11,335	9,290	14,595
